# Improving the efficiency of molecular replacement by utilizing a new iterative transform phasing algorithm

**DOI:** 10.1107/S2053273316010731

**Published:** 2016-07-15

**Authors:** Hongxing He, Hengrui Fang, Mitchell D. Miller, George N. Phillips, Wu-Pei Su

**Affiliations:** a Department of Physics and Texas Center for Superconductivity, University of Houston, Houston, Texas 77204, USA; b Department of BioSciences, Rice University, Houston, Texas 77005, USA; c Department of Chemistry, Rice University, Houston, Texas 77005, USA; d Department of Biochemistry, University of Wisconsin-Madison, Madison, Wisconsin 53706, USA

**Keywords:** molecular replacement, hybrid input–output algorithm, *ab initio* phasing, protein crystallography

## Abstract

An iterative transform method proposed previously for direct phasing of high-solvent-content protein crystals is employed for enhancing the molecular-replacement (MR) algorithm in protein crystallography. Target structures that are resistant to conventional MR due to insufficient similarity between the template and target structures might be tractable with this modified phasing method. Trial calculations involving three different structures are described to test and illustrate the methodology. The relationship of the approach to *PHENIX Phaser-MR* and *MR-Rosetta* is discussed.

## Abstract 2

An iterative transform algorithm is proposed to improve the conventional molecular-replacement method for solving the phase problem in X-ray crystallography. Several examples of successful trial calculations carried out with real diffraction data are presented.

## 1. Introduction   

Despite the success of molecular replacement (MR) as a major tool for new macromolecular structure determinations (Rossmann, 1972 ▸, 1990 ▸, 2001 ▸; Scapin, 2013 ▸), it requires a high degree of similarity between the template and target structures, a condition that is not met for many unknown structures. In particular, even for a properly placed template, there could be errors in the calculated density map that prevent further crystallographic model building.

In one approach to overcome this problem, a model rebuilding procedure has been proposed which combines both crystallographic model building (interpretation of density, likelihood of agreement with diffraction data) and energy minimization (*via Rosetta*) (DiMaio *et al.*, 2011 ▸; Terwilliger, Dimaio *et al.*, 2012 ▸) to reduce the likelihood of non-physical conformations. Such an approach has been demonstrated to increase the radius of convergence of MR. There are several other approaches to overcome this problem (Schwarzen­bacher *et al.*, 2004 ▸; Keller *et al.*, 2006 ▸; He *et al.*, 2007 ▸; Bunkóczi & Read, 2011 ▸; Brunger *et al.*, 2012 ▸; Terwilliger, Read *et al.*, 2012 ▸; Sammito *et al.*, 2014 ▸; Millán *et al.*, 2015 ▸; Carrozzini *et al.*, 2015 ▸). In this paper we consider an alternate improvement of MR based on an extension of the hybrid input–output (HIO) phasing algorithm (Fienup, 1982 ▸; He & Su, 2015 ▸). The new phasing scheme can be regarded as a powerful extension of the conventional solvent-flattening-related density modification. However, there are several important departures from the traditional approach. One is the adoption of a negative feedback function instead of the simple solvent flattening or solvent flipping for the solvent region (Wang, 1985 ▸; Leslie, 1987 ▸; Abrahams & Leslie, 1996 ▸). The other is a dynamically evolving solvent boundary (Marchesini *et al.*, 2003 ▸). For protein crystals with a high solvent content, we have shown that an accurate protein mask can be dynamically generated in an iterative transform scheme starting from a completely random initial configuration. Thus, the need for a prior knowledge of the protein mask can be eliminated, resulting in *ab initio* phasing, at least for high-solvent-content crystals (greater than about 65%).

While this new phasing method is being extended to treat more typical solvent contents, it can also be employed to supplement and enhance other existing phasing methods such as MR. To demonstrate that, we apply the methodology to several of the trial structures solved by DiMaio *et al.* (2011 ▸) that they found to be resistant to conventional MR, but were first solved by *MR-Rosetta*. Without force-field-based energy minimization, the correct phases can still be retrieved from approximately placed templates. We describe the method in detail as applied to three structures, PDB (Protein Data Bank) codes 2y92 (Valkov *et al.*, 2011 ▸), 3on5 (Joint Center for Structural Genomics, unpublished work) and 3tx8 (Brunger *et al.*, 2012 ▸), in the following sections.

## 2. Methodology   

**Figure 1:**
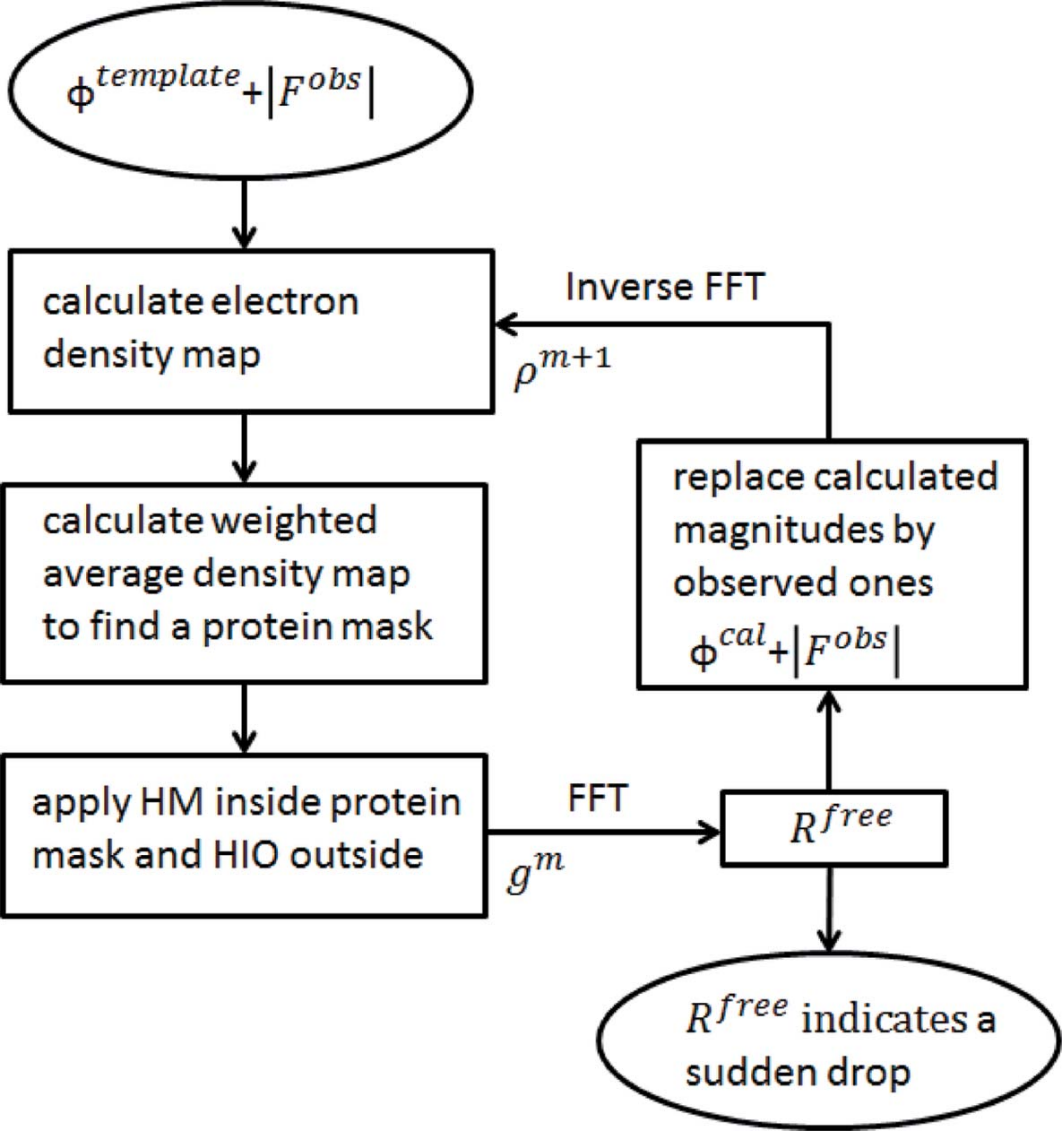
A flowchart of the iterative transform algorithm starting from the template phase estimate.

After rotation and translation with *PHENIX Phaser-MR* (McCoy *et al.*, 2007 ▸; Adams *et al.*, 2010 ▸), the template structure was placed in the unit cell. The initial phase estimate was calculated from the positioned template structure. An electron-density map was calculated using the observed Fourier magnitudes of the target crystal with the initial phases estimated from the Fourier transform of the placed template model. This type of map is generally not ready for model building due to ambiguity and lack of connectivity. Thus density modification is necessary. A weighted-average electron-density map was calculated to identify a protein mask from the calculated electron-density map. The HIO method has proved to be a very effective solvent-flattening and phase-recovery technique (Liu *et al.*, 2012 ▸; He & Su, 2015 ▸; Ayyer *et al.*, 2016 ▸) by consistently applying constraints in real space and Fourier space (Marchesini, 2007 ▸). Like real-space phasing methods (Su, 2008 ▸), the protein mask serves as a high-density support. Solvent occupies the region outside the support and the calculated electron density in the solvent region is flattened progressively *via* a negative feedback scheme. In addition to HIO, conventional histogram matching (HM) (Zhang & Main, 1990*a* ▸,*b* ▸) was also employed to modify the calculated electron density inside the protein mask. Fig. 1 ▸ shows the flowchart of the iterative method starting from phases estimated from the placed template model.

Different functions can be used as weighting functions in the calculation of the weighted-average electron-density map. The weighting function in this paper was taken to be a Gaussian described in equation (1):
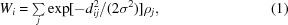
where the subscript *i* or *j* corresponds to a grid point in the unit cell. 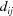 is the distance between two grid points. The radius σ can be used to control the convergence of the solvent region.

In our previous work, a cutoff value of the weighted-average density is chosen to produce a protein mask, *i.e.* grid points with a weighted-average density higher than the cutoff value are taken to be inside the protein mask. In this paper, to achieve a better final density map, we constructed a more precise protein mask by using a smaller value of radius σ in the weighting function. However, this leads to small regions of negative average density inside the protein mask. To avoid those negative pockets, we modified the calculated density by setting all the negative densities to zero before computing the weighted average.

At the beginning of the iteration process, the protein mask is an estimate and only covers part of the deposited target structure. As the iteration progresses, the calculated protein mask covers more and more of the deposited target structure due to density modification in real space and Fourier space. At the same time, the calculated electron density becomes more and more interpretable and model building becomes possible. Using a protein mask slightly larger than the deposited structure is preferred and helps to cover most of the deposited structure earlier in the iteration process. A loose protein mask including some solvent (typically less than 8% of the unit-cell volume) was adopted in our calculations. The radius σ of the weighting function started from a medium value such as 3 Å at the beginning of the iteration and decreased slowly to a smaller value, for example, 2 Å, at the end of the iteration. Such values are smaller than the ones used before in *ab initio* calculations (He & Su, 2015 ▸), because the placed template provides significantly better starting density.

A reference histogram for the protein region was calculated inside a loose protein mask of the edited template model in the target unit cell in our trials. The edited template search model was placed in the target unit cell by *PHENIX Phaser-MR*. Let 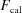 be the structure factor calculated from the atomic model of the template. A weighting function with a small radius such as 1 Å was used to calculate the weighted-average density of the electron density calculated from 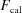. A cutoff value of the weighted-average density was adjusted to obtain a loose protein mask according to a given solvent content.

After the initial protein mask has been found, one can proceed to the calculation of the electron density inside the mask. To do that, 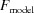 was computed from the edited template model in the target unit cell together with bulk solvent correction using *phenix.fmodel* (Adams *et al.*, 2010 ▸). The temperature factor of the bulk solvent was set to be consistent with the average temperature factor of the edited template model. If the average temperature factor of the edited template model was obviously high or low, the average temperature factor of the template model was adjusted to a more appropriate value, for example 50 Å^2^. The electron density inside the previously calculated protein mask was obtained *via* an inverse Fourier transform of 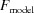.

The initial phase estimate was computed from the positioned template model in the target unit cell, which often has a protein mask similar to that of the target structure. The calculated protein mask evolved quickly to the correct position after hundreds of iterations. The template only provided an initial phase estimate. Some information about the template was lost during the iteration as the initial protein mask kept evolving.

Missing reflections in the observed data were filled in with the calculated ones and updated in each iteration cycle. The reconstructed missing reflections converged to some certain values. Measured reflections with a very small reflection angle are often not accurate and they were also replaced and updated with the calculated ones. Although our method prefers high completeness of the measured data, it still works very well when tens of measured reflections are removed. Generally, tens of reflections in the lowest-resolution shell were replaced and updated with the calculated ones during our trial calculations.

The HIO algorithm is defined in equation (2):
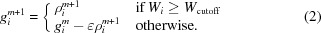
∊ is a feedback parameter which is chosen to optimize the convergence of the algorithm. It was set to be 0.9 throughout our calculations. 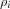 and 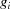 correspond to the electron-density values on the *i*th grid point before and after density modification. 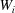 is the weighted-average density on the *i*th grid point. 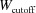 is the cutoff value of the weighted-average density.

To monitor the iteration, we calculated the mean error in the phase angle defined in equation (3),
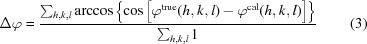
where 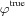 is the calculated phase of the PDB-deposited target model with bulk solvent correction and 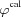 is the phase from the Fourier transform of the HIO and HM modified map.

## 3. Trial calculations   

We selected three known trial structures from those that were used in the *Rosetta* MR blind tests (DiMaio *et al.*, 2011 ▸) for our initial evaluation. These structures have also been solved and analyzed in several subsequent MR methods development studies (Brunger *et al.*, 2012 ▸; Terwilliger, Dimaio *et al.*, 2012 ▸; Terwilliger, Read *et al.*, 2012 ▸; DiMaio, 2013 ▸; Terwilliger *et al.*, 2013 ▸; Carrozzini *et al.*, 2015 ▸). All template models used in our trial calculations were the truncated and pruned models that were used for those tests and were downloaded from https://www.phenix-online.org/phenix_data/terwilliger/rosetta_2011. These are listed in Table 1 ▸.

The first trial structure was a toll-like receptor adapter (TIRAP) with PDB code 2y92 (Valkov *et al.*, 2011 ▸) comprising 145 amino acids. The protein is involved in the signal transduction, but the function is not important here. The target crystal diffracts to 3.0 Å with 77% solvent content. The lowest-resolution reflection is 38.5 Å and the completeness of the observed data is 97.5%. The search model contains 132 residues from the TIR domain of a bacterial signaling protein (PDB code 3h16; Chan *et al.*, 2009 ▸) with 86 side chains trimmed. The target and template structures share 22% sequence identity in a core of 86 residues and *SSM* (Krissinel & Henrick, 2004 ▸) aligns 104 residues with an r.m.s.d. of 2.3 Å and 12% sequence identity.

The reference histogram was the histogram of the edited template model within its loose protein mask that covered all template atoms and some solvent surrounding the template molecule, with an average density of about 0.05 e Å^−3^ above the solvent density, which was realized by adjusting the value of 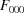. During each iteration cycle, HIO pushed the solvent density towards zero progressively and the conventional histogram matching modified the calculated electron density inside the evolving protein mask to match the reference histogram.

**Figure 2:**
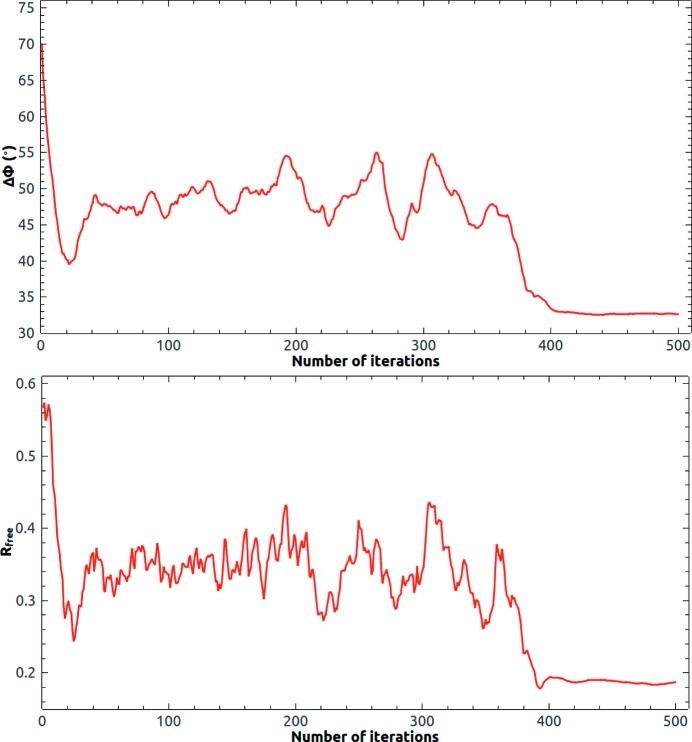
Evolution of the mean phase error and the free *R* value of 2y92 at 3.0 Å resolution starting from the template phase estimate. HIO was gradually turned off after 200 iteration cycles.

The initial phase estimate was computed from the template model after being placed into the target unit cell by *PHENIX Phaser-MR*. Missing reflections in the observed data were filled in and updated with the corresponding calculated ones in each iteration cycle. The resolution cutoff used in the iteration was somewhat higher than the resolution cutoff of the observed data. The mean error in the phase angle defined in equation (3) and the free *R* value (Brünger, 1992 ▸) were calculated to monitor the iteration. About 2% of the observed reflections were randomly selected for the calculation of 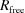. Free reflections were replaced with calculated values as for missing reflections. Fig. 2 ▸ shows the evolution of the mean error in the phase angle and the free *R* value. The initial mean error in the phase angle was 70° and it dropped to 40° in 20 iterations and became stable. Generally, the value of the HIO-modified density in the solvent region is unlimited. We found if this value was limited to within a certain maximum value HIO performed better. The maximum value of the HIO-modified density was limited to ±1.0 e Å^−3^ at the beginning of all trial calculations. In other words, if the HIO-modified density was greater than 1.0 e Å^−3^, 1.0 e Å^−3^ would be used. If the HIO-modified density was less than −1.0 e Å^−3^, −1.0 e Å^−3^ would be used. For 2y92, the allowed maximum value of the HIO-modified density gradually decreased from ±1.0 e Å^−3^ at the 200th iteration to ±0.01 e Å^−3^ at the 400th iteration. HIO was slowly turned off in 200 iterations. In the last 100 iteration cycles, the allowed maximum value of the HIO-modified density in the solvent region was limited to a small empirical value, for example, ±0.01 e Å^−3^. Because the true electron density in the solvent region is not exactly a constant, compared with traditional solvent flattening, limited HIO density modification often noticeably improves the calculated phases and leads to a smaller free *R* value. The mean phase error further dropped to 32°. Notice that the drops in 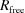 tracked the drops in 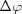 very well.

**Figure 3:**
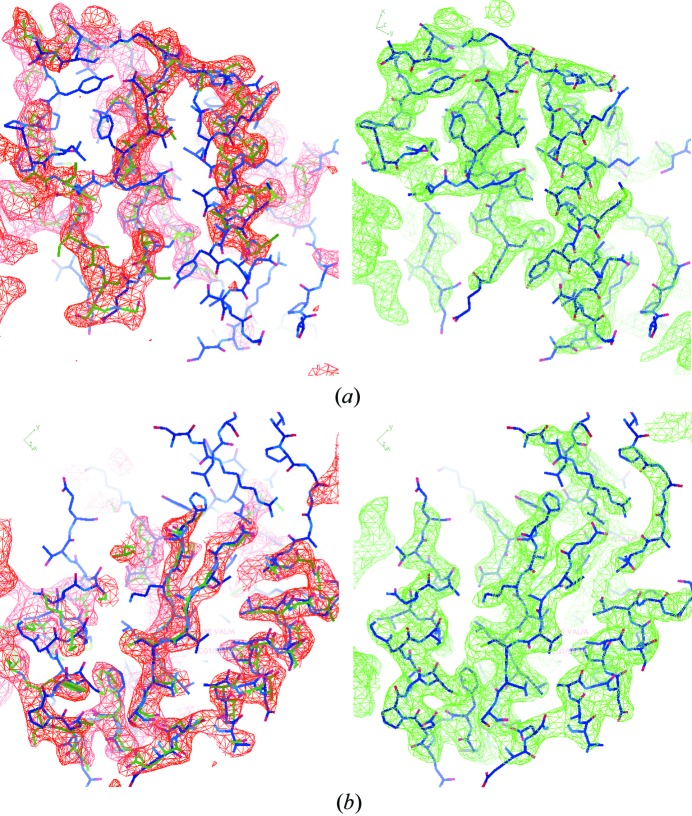
The initial and the final calculated electron-density maps of 2y92 at 3.0 Å resolution with the PDB-deposited structure superimposed. The initial density map is in red in the left panels and the final density map is in green in the right panels. The blue wireframe is the target structure. The green wireframe in the left panel is the template model. The maps were generated with *Coot* (Emsley *et al.*, 2010 ▸).

The initial electron-density map calculated from the initial phase estimate and the observed Fourier magnitudes of the target crystal is shown in red in the left panels of Fig. 3 ▸. The initial map is ambiguous and is not connected in certain regions. When the target and template structures were superimposed on the map, the density map deviated significantly from the target in certain secondary structure regions and more closely matched the template structure. Conventional MR fails as it is not easy to rebuild the model and fit it into this map. Compared with the initial density map, the final map, shown in green in the right panels of Fig. 3 ▸, has been improved so much that it can be used directly for model building. Most of the bias in the initial density map has been corrected. The ambiguity disappears and the connectivity becomes very clear. When we superimposed the target structure onto the final density map, they matched very well. There are small parts of the target structure that remained outside the density in the final map due to the final phase error. The final density map was used to automatically build a model with the model building software *ARP/wARP* (Langer *et al.*, 2008 ▸). About 85% of the sequence could be successfully placed.

The correlation coefficients (CCs) between the calculated density map and the deposited target structure as well as with the placed template structure were calculated with the command-line tool *phenix.get_cc_mtz_pdb* (Adams *et al.*, 2010 ▸). At the beginning of the iteration, the CC value to the deposited target structure was 0.38, indicating limited agreement between the initial density map and the deposited target structure. However, at the end of the iteration, the value of CC increased to 0.78, which indicated very good agreement between the final density map and the deposited target structure. Likewise, for the placed template structure the starting map CC was 0.76 while the CC to the final map was 0.39, which is evidence that the bias from the template structure has been reduced in the final map relative to the map after MR.

**Figure 4:**
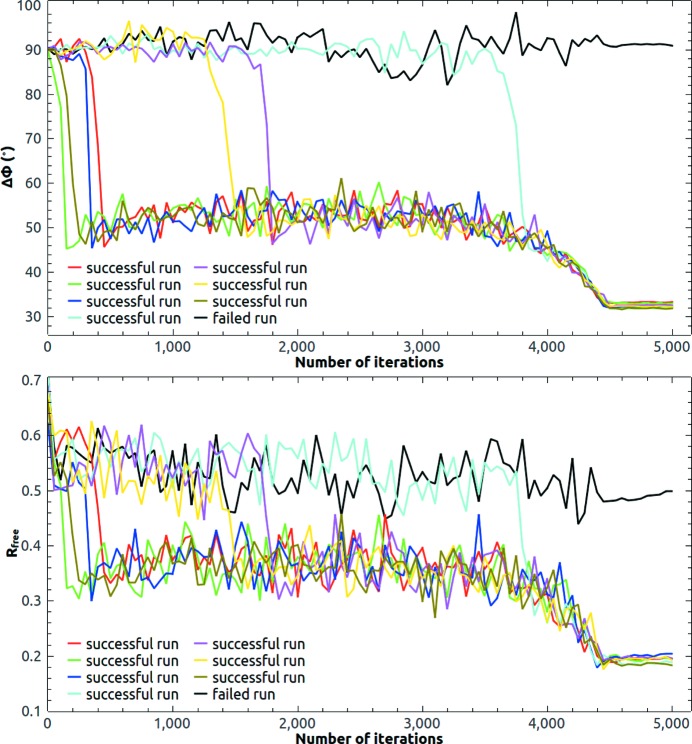
Evolution of the mean phase error and the free *R* value of 2y92 at 3.0 Å resolution starting from random phases. There were seven successful runs among ten attempts. A sudden drop of 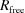 indicates a convergent run.

**Figure 5:**
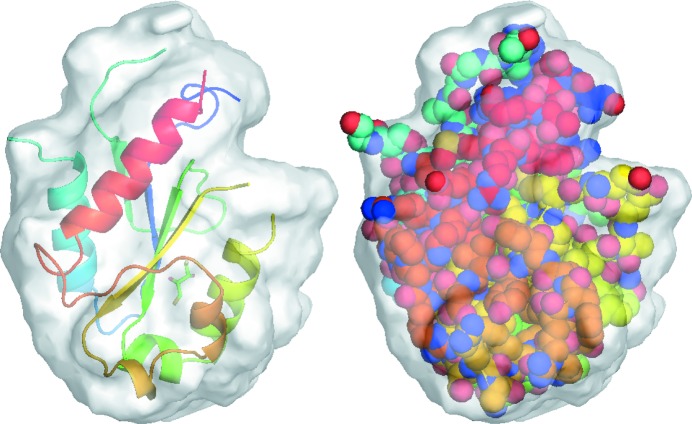
The final calculated protein mask of 2y92 at the end of a successful run starting from random phases. The PDB-deposited structure of 2y92 is superimposed which is displayed in cartoons in the left panel and in spheres in the right panel. The figure was generated with *PyMOL* (Schrödinger, LLC).

Due to the large solvent content (77%), it is possible to phase the crystal of 2y92 directly from random phases, but it takes more iterations to make the protein mask evolve to the correct shape and position. Fig. 4 ▸ shows eight runs starting from random phases. It took about 1000 iterations on average to obtain an interpretable density map for a convergent run indicated by a sudden drop of the free *R* value, while 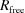 almost did not change for a failed run. HIO was gradually turned off after 3500 iteration cycles. In the last 500 iteration cycles, the allowed maximum value of the HIO-modified density in the solvent region was limited to ±0.01 e Å^−3^. The final mean phase error is about 32°, which is the same as the final mean phase error starting from the template estimate. During the iterations, the radius σ of the weighting function was varied from 4 to 2 Å. The choice of a larger value of σ (He & Su, 2015 ▸) would result in a slightly larger phase error. Fig. 5 ▸ shows the final calculated protein mask at the end of a successful run starting from random phases. The calculated protein mask covers the deposited structure of 2y92 very well. If the solvent content becomes less, such as 62% in the next trial structure, it is difficult to phase directly from a random start. A good template is required in that case.

The second trial structure was a xanthine dehydrogenase from *Bacillus halodurans* with PDB code 3on5 (Joint Center for Structural Genomics, unpublished work), with 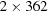 amino acids. The crystal diffracts to 2.8 Å, with the lowest reflection at 45.2 Å. The completeness of the data is 99.8%. The solvent content is 62% which makes an *ab initio* phasing scheme difficult and a good template is necessary. The starting model was the *phenix.automr* search solution that placed two copies of the 145 amino-acid C-terminal domain followed by two copies of the 165 amino-acid N-terminal domain of PDB code 2we8 chain A (H. J. Cho & B. S. Kang, unpublished work) and which is the starting point for the downloaded *phenix.mr_rosetta* test script (DiMaio *et al.*, 2011 ▸). The sequence identity is 29%, which is somewhat lower than the threshold where conventional MR works reliably. Comparing the similarity of the individual MR template search domains with *SSM* produces alignments of 109–123 residues per domain with core r.m.s.d.’s of 1.9–2.6Å.

**Figure 6:**
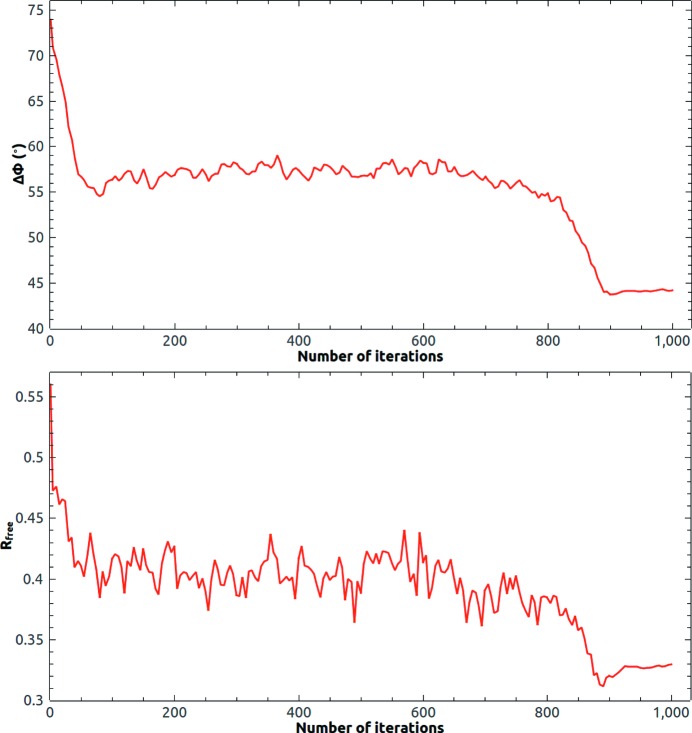
Evolution of the mean phase error and the free *R* value of 3on5 at 2.8 Å resolution starting from the template phase estimate.

The reference histogram was again calculated from the template within a loose protein mask. About 200 measured reflections in the lowest-resolution shell were updated with the calculated ones. The initial phase estimate was calculated from the positioned template model in the target unit cell and the evolution of the mean phase error and free *R* value is depicted in Fig. 6 ▸. The mean error in the phase angle was about 74° at the beginning. It dropped to about 55° in 80 iteration cycles and became stable. At the end of the iteration process, it further dropped to 44° as HIO was gradually turned off. In the last 100 iteration cycles, the allowed maximum value of the HIO-modified density in the solvent region was limited to ±0.02 e Å^−3^.

**Figure 7:**
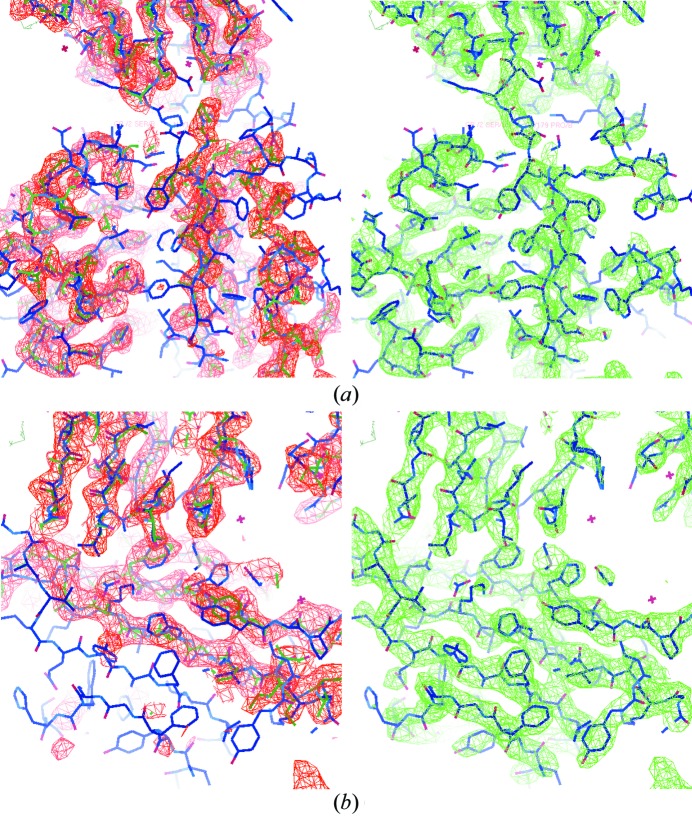
The initial and the final calculated electron-density maps of 3on5 at 2.8 Å resolution with the PDB-deposited structure superimposed. The initial map is in red in the left panels and the final map is in green in the right panels. The target structure is superimposed as the blue wireframe. The template model is shown as the green wireframe in the left panels.

The initial density map of 3on5 is depicted in red in the left panels of Fig. 7 ▸. The CCs of the initial density map with the deposited target structure as well as with the placed template structure were 0.41 and 0.78, respectively. Despite the large phase error and the fact that 3on5 is a larger structure than 2y92, the final calculated density map (shown in green in the right panels of Fig. 7 ▸) is very good with a CC value of 0.80 to the deposited target structure and a CC value of 0.34 to the placed template structure. About 80% of the sequence was successfully placed by the automated model building procedure *ARP/wARP* (Langer *et al.*, 2008 ▸). The twofold non-crystallographic symmetry of this structure has not been exploited in the phasing process.

**Figure 8:**
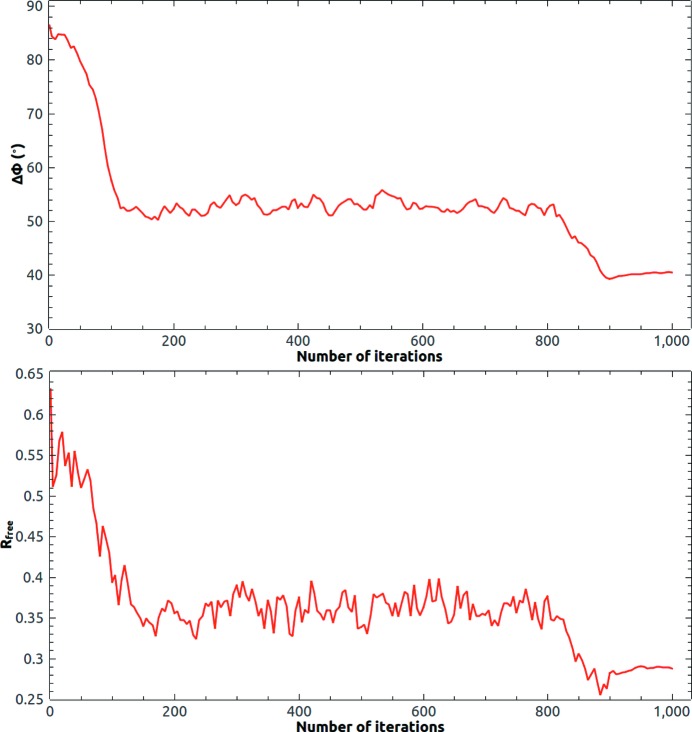
Evolution of the mean phase error and the free *R* value of 3tx8 at 2.97 Å resolution starting from the template phase estimate.

The final trial structure was a putative succinyl-diamino­pimelate desucccinylase from *Corynebacterium glutamicum* with PDB code 3tx8 (Brunger *et al.*, 2012 ▸). The resolution of the diffraction data ranges from 2.97 to 29.6 Å with a completeness of 98.7%. The solvent content of the crystal is 72%. The *Phaser*-placed template of DiMaio *et al.* (2011 ▸) included 352 residues from PDB code 1vgy (Badger *et al.*, 2005 ▸) with 218 trimmed side chains. *SSM* aligns a core of 318 C_α_ pairs with 2.2 Å r.m.s.d. The sequence identity (20%) is the lowest and the starting phase error (86°) is the highest among the three trial structures. Despite that, a small mean phase error (40°) was reached at the end of the calculation. During the calculation, about 60 measured lowest-resolution reflections were updated with the calculated ones. Fig. 8 ▸ shows the evolution of the mean phase error and the free *R* value. The mean phase error dropped from 86° to 52° in 120 iterations and became stable. HIO was progressively turned off after 500 iteration cycles. In the last 100 iteration cycles, the allowed maximum value of the HIO-modified density in the solvent region was limited to ±0.01 e Å^−3^ and the final mean phase error was about 40°.

**Figure 9:**
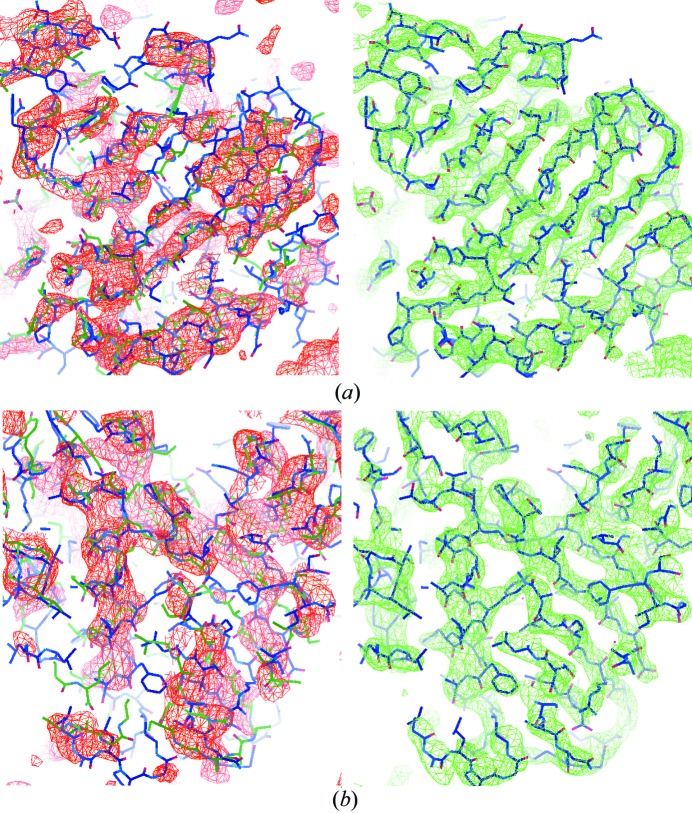
The initial and the final calculated electron-density maps of 3tx8 at 2.97 Å resolution with the PDB-deposited structure superimposed. The initial density map is in red in the left panels and the final density map is in green in the right panels. The target structure is superimposed as the blue wireframe. The template model is shown as the green wireframe in the left panels.

The initial electron-density map of 3tx8 is shown in red in the left panels of Fig. 9 ▸ with a CC value of 0.31 to the deposited target structure and a CC value of 0.68 to the placed template structure. The initial map shows ambiguity in some regions where it does not cover the deposited target structure. The final electron-density map shown in green in the right panels of Fig. 9 ▸ has a CC value of 0.77 to the deposited target structure and a CC value of 0.31 to the placed template structure. About 80% of the sequence could be successfully placed by the automated model building software *ARP/wARP* (Langer *et al.*, 2008 ▸).

**Figure 10:**
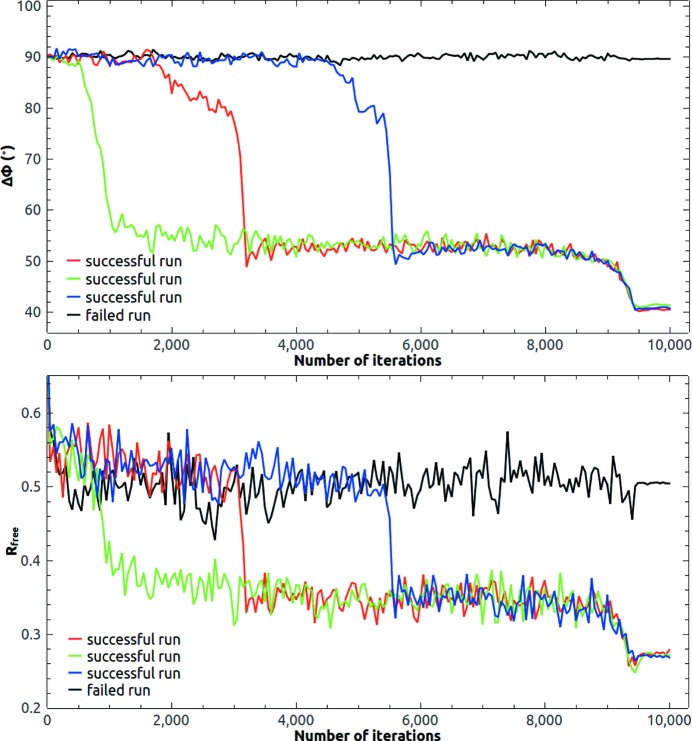
Evolution of the mean phase error and the free *R* value of 3tx8 at 2.97 Å resolution starting from random phases. There were three successful runs among 15 attempts starting from random phases. A sudden drop of 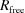 indicates a convergent run.

**Figure 11:**
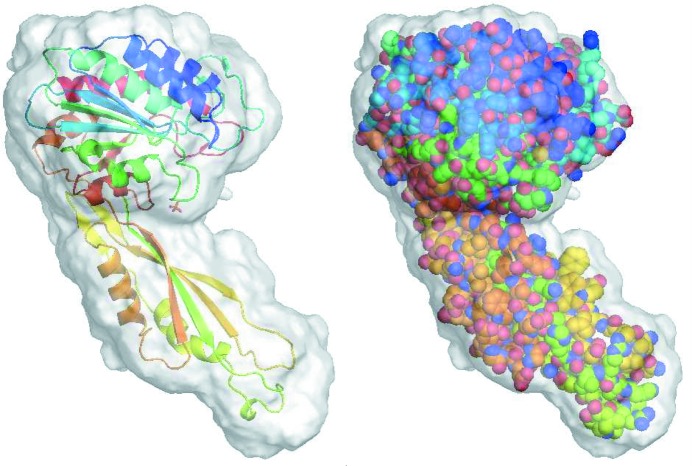
The final calculated protein mask of 3tx8 at the end of a successful run starting from random phases. The deposited structure of 3tx8 is superimposed which is displayed in cartoons in the left panel and in spheres in the right panel.

The solvent content of 3tx8 is large enough for *ab initio* phasing (He & Su, 2015 ▸). Starting from random phases, we got three successful runs among 15 attempts. Since the initial map was random, the radius σ of the weighting function started from 4 Å at the beginning of the iteration and decreased slowly to 2 Å at the end of the iteration. The reference histogram was calculated from the template structure. The evolution of the mean phase error and the free *R* value is shown in Fig. 10 ▸. Fig. 11 ▸ shows the final calculated protein mask at the end of a successful run. The final calculated protein mask matches the deposited structure of 3tx8 quite well.

## 4. Discussion   

The success of the MR method depends significantly on the similarity between the target and template structures (Scapin, 2013 ▸; Schwarzenbacher *et al.*, 2004 ▸). As sequence identity between the target and template structures decreases, the increased likelihood of significant structural differences (Chothia & Lesk, 1986 ▸; Gan *et al.*, 2002 ▸; Krissinel, 2007 ▸) makes finding an MR solution more difficult. Even when a distant homolog can be placed, the poor quality of the starting map prevents rebuilding and refinement. For example, when the sequence identity is between 30% and 40%, it is usually possible to solve the phase problem using *Phaser-MR*, but sometimes it is more difficult. If the sequence identity is between 20% and 30%, it usually becomes difficult to retrieve the phase using *Phaser-MR*. If it is possible then one needs to search and prepare a proper model carefully. *MR-Rosetta* has made further progress towards MR with lower sequence similarity templates. It is usually possible to solve the target structure when the sequence identity is between 20% and 30%, but it still requires careful model search and preparation. When the sequence identity is below 20%, *Phaser-MR* is unlikely to work and it is difficult for *MR-Rosetta* to work, if at all possible. We have provided a supplementary approach which may be critically needed for MR when the sequence identity is below 30%.

It is clear from our trial calculations that, for high-solvent-content protein crystals, the HIO iterative transform phasing can be employed to significantly enlarge the radius of convergence of conventional MR. Without doing any energy minimization, we have achieved basically the same results as *MR-Rosetta*. Since it does not involve model rebuilding, our approach is very simple conceptually. It does suffer from the disadvantage of being incapable of dealing with low-solvent-content crystals at present. Also, the template needs to be approximately (if not accurately) placed for HIO. *Phaser-MR* can help with the placement. An accurately placed template is preferred but not strongly required. Take 3on5 as an example. One can place the template by hand to make it approximately overlap with the target structure in the unit cell. The initial phase estimate had a mean phase error of about 86°, which required more iteration cycles (about 200) in our test to reach convergence (data not shown). The final phase error was the same as the one starting from an accurately placed template.

For medium (close to 50%) solvent content, if non-crystallographic symmetry (NCS) exists, the iterative transform/projection algorithm works well with some initial phase information such as a low-resolution molecular envelope and the position of the non-crystallographic axes (Millane & Lo, 2013 ▸; He & Su, 2015 ▸; Lo *et al.*, 2015 ▸). It works best when the exact position of the non-crystallographic axis is known which is not easily acquired before model building. If NCS does not exist, the HIO algorithm in its current form is not yet applicable for direct phasing of crystals with a solvent content close to 50%, but as we have seen in the trial calculations, the existence of a template can lead to a solution. An implementation including both HIO and *MR-Rosetta* would presumably be a more powerful refinement tool than either one alone. For example, a difficult structure may require *MR-Rosetta* to properly modify and place a template. The placed template may still not be good enough for model building, yet it might be successfully refined through HIO.

A natural question is how this work fits in to the existing crystallographic methods. To assess how maps from the HIO iteration compare with those obtainable from several other common density-modification schemes, we ran trials using *Solomon* (Abrahams & Leslie, 1996 ▸), *DM* (Cowtan, 1994 ▸), the prime-and-switch method (Terwilliger, 2004 ▸) using *phenix.autobuild* and *phenix.resolve*, *Pirate* (Cowtan, 2000 ▸) and *Parrot* (Cowtan, 2010 ▸). As pointed out by Bunkóczi *et al.* (2015 ▸), such comparisons are inherently biased as we are better at optimizing our own code than that of others. We sought to mitigate this bias by testing runs with multiple solvent contents, starting maps and program parameters around the defaults, but ultimately our results are a lower bound for the phase improvement from these methods. Starting and ending maps were compared with the 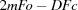 maps of the deposited target models optimized by the *PDB_REDO* server (Joosten *et al.*, 2014 ▸) using *phenix.get_cc_mtz_mtz*. Depending on which starting map was used, the starting correlations were 0.47–0.56 for 2y92, 0.44–0.59 for 3on5 and 0.34–0.49 for 3tx8. The HIO iteration resulted in maps with correlations of 0.85–0.88 for all three test cases. For 2y92 there were several other methods with final map correlations exceeding 0.8 including *Parrot*, prime-and-switch and *DM*, with the remaining methods finishing around 0.7. For 3on5, prime-and-switch was the only other method to exceed 0.8 and *Pirate* was able to exceed 0.7. The 3tx8 target proved to be more challenging and none of the other methods exceeded a map correlation of 0.65 in our trials.

In summary, the HIO method led to maps with a correlation greater than 0.8 to the *PDB_REDO* 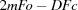 maps for all of these cases. While the other methods tested worked for some cases, all had trouble with at least one of these cases. The optimal algorithm is likely sample and crystal-form dependent. We see evidence of this even in our limited testing where we saw different rankings in the order of the program successes. Researchers benefit by having multiple complementary methods available for phase improvement as each has cases where it excels. There are several very good methods, some of which may be better suited to a subset of cases than others and it is useful to have multiple options to find a solution in difficult cases. HIO is a method that complements the existing methods of density modification and is very strong for crystals with high solvent contents.

## 5. Conclusion   

The HIO iterative phasing approach is capable of improving the MR method when the sequence identity between the target and the template structures is below 30%. The MR-placed template structure provides an initial phase estimate and an initial protein mask. It is simple and straightforward to apply this approach and it requires less computing resources than *MR-Rosetta*. Our calculations typically took less than 1 h on a standard laptop computer. Because no model building is involved during the iteration and the molecular mask evolves, the final density map has less model bias. The results of our trial calculations show that the final density map is ready for automated model building.

We have tried several structures and have obtained good results as shown here. Source code is available from the authors upon request. More trial calculations involving structures with lower solvent contents and other variations are clearly desirable.

## Figures and Tables

**Table 1:** Trial calculations involving three target structures

							Overall correlation coefficient					
							Placed template model		Deposited target model		Initial	Final
PDB code	Space group	Sequence identity (%)	No. of amino acids	No. of NCS copies	Solvent content (%)	Resolution (Å)	Initial map	Final map	Initial map	Final map	mean phase error (°)	mean phase error (°)
2y92	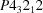	22	145	1	77	3.01	0.76	0.39	0.38	0.78	70	32
3on5	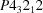	29	362	2	62	2.80	0.78	0.34	0.41	0.80	74	44
3tx8	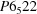	20	369	1	72	2.97	0.68	0.31	0.31	0.77	86	40
